# The Usefulness of AirSeal™ Intelligent Flow System in Gas Insufflation Total Endoscopic Thyroidectomy

**DOI:** 10.1007/s12070-022-03257-0

**Published:** 2022-11-06

**Authors:** Hiroshi Katoh, Yoshifumi Ikeda, Yoshiyuki Saito, Mitsuo Yokota, Mariko Kikuchi, Norihiko Sengoku, Kaoru Fujisaki, Takafumi Sangai

**Affiliations:** 1 Department of Breast and Endocrine Surgery, Kitasato University Hospital, 1-15-1 Kitasato, Minami-Ku, Sagamihara, Kanagawa 252-0374 Japan; 2 Department of Surgery, International University of Health and Welfare, Atami Hospital, Atami, Japan; 3 Department of Surgery, Keio University School of Medicine, Tokyo, Japan

**Keywords:** AirSeal intelligent flow system, Endoscopic thyroidectomy, Gas insufflation thyroidectomy, Minimally invasive thyroidectomy, Total endoscopic thyroidectomy

## Abstract

Total endoscopic thyroidectomy (TET) using low CO_2_ insufflation provides cosmetic advantage, excellent working space and visibility. On the contrary, suctioning blood or mist/smoke produced by energy device application causes narrowing of working space especially in neck surgery. In this regard, AirSeal intelligent flow system would be particularly suitable in TET. However, the benefit of AirSeal is unknown in TET unlike abdominal surgery. Therefore, the impact of AirSeal was evaluated in TET in this study. Twenty patients who underwent total endoscopic hemithyroidectomy were retrospectively analyzed. Insufflation was conducted by either conventional or AirSeal system according to the surgeon’s preference. Short-term surgical outcomes including operation time, bleeding, frequency of scope cleaning, and disappearance of subcutaneous emphysema were compared as well as actual visibility. AirSeal application dramatically reduced obstacle smoke/mist and prevented narrowing working space by suctioning. Frequency of scope cleaning was significantly less in AirSeal group than that in conventional group (*p* = 0.016). In patients with nodule < 5 cm, intraoperative hemorrhage was less in AirSeal group than that in the counterpart (*p* = 0.077) regardless of larger nodule size in AirSeal group (*p* = 0.058). Notably, subcutaneous emphysema around surgical cavity disappeared significantly earlier in AirSeal group than in the counter parts (*p* = 0.019). On the contrary, AirSeal application did not shorten operation time in the current study. AirSeal provided excellent visibility and seamless operation. AirSeal has great potential to decrease not only surgeon’s stress but surgical invasion on patients. The results in this study give rational to AirSeal application to TET.

**Supplementary Information:**

The online version contains supplementary material available at 10.1007/s12070-022-03257-0.

## Introduction

Although an endoscopic surgery was described with relative delay in thyroid diseases compared to other organs because of the narrow operative field, cosmetic advantages of endoscopic thyroidectomy are inarguably accepted [1]. After video-assisted neck surgery (VANS), a gasless endoscopic surgical approach, was described [2], VANS method has been widely applied. The VANS method allows manual palpation of the tumor from the chest wall incision, and excellent bleeding control. On the other hand, total endoscopic thyroidectomy (TET) by the axillary approach or else enables high degree of freedom in surgical incision that leads to further cosmetic advantage [3–5] than VANS method. Low pressure CO_2_ gas insufflation provides excellent working space and hemostatic effect on minor bleeding, contributing to excellent visibility. These are also great advantages of TET compared to a gasless VANS method. However, suction of blood or mist/smoke produced by energy device use causes narrowing of working space in TET, which may lead to extended operation time or unexpected complication due to poor visibility. Accordingly, ensuring stable working space is required to maximize the advantages of TET. AirSeal™ intelligent flow system (SurgiQuest, Milford, USA; ConMed Japan, Tokyo) is an insufflation system that can maintain a fixed pressure in surgical field by continuously monitoring and adjusting CO_2_ flow rates. AirSeal system consists of a contiguous trilumen filter tube set that connects to one valveless access port. AirSeal system allows immediate response to slight changes in the set pressure by automatically adjusting flow rate in real time. Such immediate and automatic compensation of CO_2_ pressure and volume loss provides great benefit in reducing effects on working space by intraoperative suctioning. AirSeal system is widely used in abdominal surgery especially in robotic surgery [6–9]. Taking the narrower space of neck into account, AirSeal system would provide great advantages in visibility of TET further than abdominal surgery. Therefore, we aimed to clarify whether AirSeal system can provide benefits in TET in this study.

## Patients and Methods

We evaluated consecutive 20 patients who underwent endoscopic hemithyroidectomy at Kitasato University Hospital from January 2017 to December 2019. Preoperative diagnosis for hemithyroidectomy was as follows: 15 patients, adenomatous nodule(s); 3 patients, follicular tumor; 1 patient, papillary thyroid cancer; 1 patient, intrathyroidal parathyroid adenoma (Table 1). TET was performed by one surgeon similarly as previously described [10–12]. Briefly, a 20–30 mm incision was placed in the axilla or the lateral anterior chest. After making working space under the platysma muscle by blunt dissection and inflation of a PDB™ round balloon (Medtronic), either two 5 mm trocars (without AirSeal) or one 5 mm trocar and AirSeal port (with AirSeal) were placed through XS-size FREE ACCESS™ (TOP, Tokyo) fixed to the outer ring of a XS-size SMART RETRACTOR™ (TOP) that was inserted through the skin incision. Carbon dioxide (CO_2_) was insufflated to 6–8 mmHg. An additional 5 mm trocar was inserted supraclavicular and near the incision. HARMONIC HD 1000i (36 mm) (Ethicon Japan) was applied as an energy device, and the seven-mode was used to seal superior/inferior thyroid arteries. The anterior border of the sternocleidomastoid muscle was dissected from the sternohyoid muscle, then the omohyoid muscle was dissected. The sternothyroid muscle was dissected from the sternohyoid muscle and the working space was made for subsequent hemithyroidectomy. The information on operation including (endoscopic) operation time, blood loss, and frequency of scope cleaning were evaluated (Table 1).

**Table 1:** Patient demographics taking into account application of AirSeal system

**Air seal**	Age	Gender	Preoperative diagnosis	Nodule size (mm)	Surgery	Blood loss (ml)	operation time (min)	Endoscopic operative time (min)	Frequency of scope cleaning	Frequency of scope cleaning/endoscopic operative time (hr)	Disappearance of subcutaneous emphysema (day)
**No**	42	F	AN	32	RHT	20	169	134	16	7.2	4
**No**	40	F	PTC	15	RHT, CND	8	178	133	11	5.0	3
**No**	13	F	AN	33	LHT	10	175	129	12	5.6	4
**No**	57	F	PHPT (intrathyrod)	15	RHT	65	182	137	14	6.1	3
**No**	57	F	AN	25	LHT	10	241	196	24	7.3	2
**No**	51	F	AG	43	LHT	50	168	133	8	3.6	4
**No**	36	M	AG	70	LHT	10	166	131	8	3.7	5
**No**	30	F	AG	45	RHT	slight	170	140	4	1.7	6
**No**	28	F	AN	51	LHT	slight	207	161	9	3.4	7
**Yes**	47	F	AN	35	RHT	slight	166	121	9	4.5	3
**Yes**	31	F	FT	28	RHT	slight	187	142	11	4.6	2
**Yes**	29	F	FT	37	LHT	12	240	195	9	2.8	4
**Yes**	54	F	AN	50	RHT	71	191	146	6	2.5	3
**Yes**	38	F	AN	60	RHT	slight	176	146	9	3.7	3
**Yes**	53	F	AN	47	RHT	slight	172	131	7	3.2	3
**Yes**	53	F	AN	44	RHT	50	172	138	4	1.7	3
**Yes**	46	F	AN	45	RHT	7	173	136	3	1.3	2
**Yes**	34	F	FT	40	RHT	slight	169	144	3	1.3	2
**Yes**	44	F	AN	59	RHT	slight	252	222	5	1.4	3
**Yes**	23	F	AN	46	LHT	slight	257	218	6	1.7	3
p*	n.s			0.181		0.161	n.s.(0.407)	n.s.(0.309)	0.016	0.006	0.019

*AN* adenomatous nodule(s), *PTC* papillary thyroid carcinoma, *PHPT* primary hyperparathyroidism, *FT*, follicular tumor, *RHT* right hemithyroidectomy, *LHT* left hemithyroidectomy, *CND* central neck dissection

*t test

This study was approved by the ethics committee (IRB) of the Kitasato University School of Medicine (the IRB approved #B22-004), and was performed in accordance with the clinical research guidelines of the IRB of the Kitasato University School of Medicine. All individuals gave written informed consent for pathologic assessment and routine blood sample analyses on their samples, and clinical data. The study was conducted in accordance with the Declaration of Helsinki (as revised in 2013).

Mann–Whitney’s U test was used to compare continuous variables. A *p* value < 0.05 was considered significant. All statistical analyses were conducted using a JMP Pro14 (SAS Institute, Cary, NC).

## Results

There was no significant difference in preoperative patient characteristics (Table 1). Nodule sizes were marginally larger in AirSeal group without statistical significance (p=0.181). Average nodule sizes were 36.6 ± 5.9 (s.e.m.) mm and 44.6 ± 2.9 mm in patients without AirSeal and with AirSeal, respectively.

**Fig. 1:**
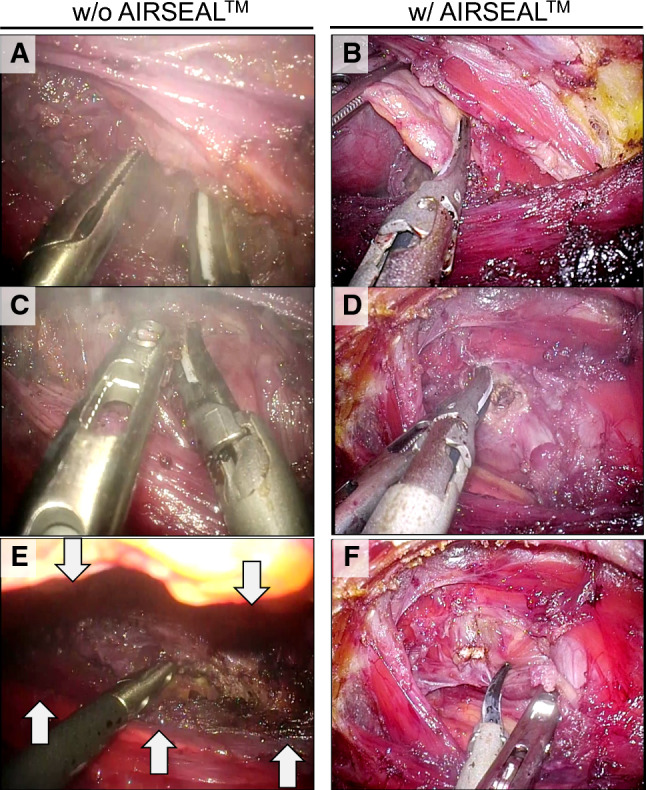
Representative images of total endoscopic right hemi-thyroidectomy taking into account AirSeal application. Smoke/mist caused by energy device (Harmonic HD) obstructs visibility in non-AirSeal used conventional group (**A** and **C**). AirSeal application dramatically improved visibility by decreasing smoke/mist (**B** and **D**). Suctioning of smoke/mist narrows working space in non-AirSeal group (**E**). AirSeal system prevents narrowing on suctioning by retaining stable pressure (**F**). Video presentations demonstrate excellent visibility by AirSeal use

**Fig. 2:**
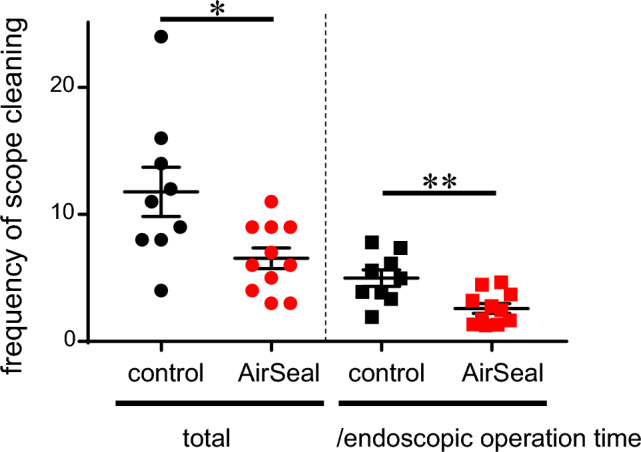
Frequency of scope cleaning was reduced by AirSeal application. AirSeal system significantly reduced frequency of scope cleaning. Left, total frequency per an operation. Right, frequency corrected by endoscopic operation time (hr). *p < 0.05, **p < 0.01

CO_2_ insufflation was performed at 6 mmHg in most of cases of AirSeal group (8/11) and 6 mmHg pressure was sufficient for working space. On the contrary, 8 mmHg insufflation was required for all of cases of conventional group to obtain enough working space. Application of AirSeal system dramatically reduced smoke and mist caused by an energy device, providing excellent visibitily (Fig. 1A–D, Supplementary videos 1 and 2). On suctioning mist/smokes produced by an energy device, AirSeal system prevented narrowing working space and greatly contributed to keep wide and clear visibility, leading to seamless operation (Fig. 1E, F, Supplementary videos 1 and 2). Indeed, AirSeal application significantly decreased frequency of scope cleaning from 11.8 ± 1.9 times to 6.5 ± 0.8 times per operation (p=0.016, Table 1, Fig. 2 left). Frequency of scope cleaning corrected by endoscopic operation time (hr) further showed remarkable improvement by AirSeal system which reduced the cleaning frequency during endoscopic procedure from 4.8 ± 0.6 times to 2.6 ± 0.4 times (*p* = 0.006, Fig. 2 right). AirSeal application only marginally contributed to reduction of intraoperative blood loss (19.2 ± 7.6 ml in non−AirSeal group vs. 12.7 ± 7.4 ml in AirSeal group, *p* = 0.161). In patients with nodule size < 5 cm, AirSeal decreased intraoperative blood loss from 23.3 ± 9.2 ml to 8.6 ± 6.1 ml (*p* = 0.077) regardless of larger nodule size (40.3 ± 2.3 cm in AirSeal group vs. 29.7 ± 4.6 cm in non−AirSeal group, *p* = 0.058). Notably, time to disappearance of subcutaneous emphysema around surgical cavity was significantly shorter in AirSeal group (2.82 ± 0.18 days) than in the counter parts (4.22 ± 0.52 days) (*p* = 0.019). On the other hand, total operation time and endoscopic operation time were not improved by AirSeal application in the current study.

## Discussion

In the present study, clinical impact of AirSeal intelligent flow system was first evaluated in total endoscopic thyroidectomy (TET). We report that AirSeal application dramatically improves visibility and further suggested possibility to decrease surgical stress on patients.

Recently, AirSeal system have been widely applied in laparoscopic or intraabdominal robotic surgery since Herati et al. first clinically evaluated in urologic laparoscopic surgery [13]. The hallmark of AirSeal system is its control unit which comprises a triple lumen filter tube set connected to one valveless AirSeal trocar. CO_2_ inflow is supplied from one lumen of the filter tube set, the second lumen provides CO_2_ outflow, and real−time monitoring and compensation of pressure in the surgical space is performed via the third lumen. In laparoscopy and intraabominal robitic surgery, such delicate pressure control has allowed a stable pneumoperitoneum and excellent visibility which leaded to reduction of blood loss, and further decreased laparoscopy−associated shoulder pain [6–9, 14, 15]. In TET, the insufflated operating field is much smaller than abdominal surgery. Because of the smaller working space, less smoke/mist produced by energy device easily obstruct the view and suctioning can further cause narrowing space. Therefore, AirSeal application to TET can contribute to improvement of visibility more than to abdominal surgery. Indeed, AirSeal use dramatically improved visibility in patients who underwent TET in our study (Fig. 1 and Video presentation), statistically confirmed by reduction of scope cleaning frequency (Table 1 and Fig. 2). In addition, intraoperative blood loss was decreased by AirSeal use although it is not statistically significant. On the other hand, contribution to shortening operation time has been still contradictory [14, 16]. Similarly, AirSeal application did not improve both total and endoscopic operation time in this study (Table 1).

Of note, subcutaneous emphysema around surgical cavity disappeared significantly earlier in AirSeal group, maybe because 6 mmHg CO_2_ insufflation was sufficient in most of patients by AirSeal application compared to 8 mmHg in conventional group. This result is consistent with a previous report in pediatric laparoscopic appendectomy [17]. Sroussi et al. reported that lower pressure resulted in less postoperative shoulder pain in gynecological laparoscopy [18]. These results suggest that AirSeal not only improve visibility but surgical invasion on patients. The valveless trocar allows to release excessive gas on the unexpected pressure elevation as a pop−off valve. This may also explain earlier disappearance of subcutaneous emphysema around surgical cavity. Previous studies reported that the valveless trocar system reduces CO_2_ absorption during laparoscopy by keeping intraabdominal pressures within the acceptable range longer than the conventional system [16, 19]. Additionally, Galizia et al. alerted that pulsatile flow fluctuation in conventional CO_2_ insufflators affects vessel tone regulation, triggering hypoperfusion and reperfusion injury, inducing oxidative stress, cellular injury, and organ dysfunction in laparoscopy [20].

Along with reducing smoke, the filter component of the AirSeal tube set minimizes the hazards of surgical smoke by removing carcinogens and pathogens from smoke down to 0.01 μm [16]. This is great advantage of AirSeal system especially in COVID−19 pandemic as well.

The limitation of this study is that this report is based on retrospective setting in small number of patients in one institution. Future studies from case−matching its cohorts and larger, multi−center, randomized control trials are needed to demonstrate the impact of AirSeal system on intraoperative and postoperative patient outcomes.

In conclusion, AirSeal system provides great benefits in visibility in TET, leading to seamless procedure and mitigating surgeon’s stress. AirSeal may have benefits in surgical invasion on patients as well. Further investigation would define the clinical impact of AirSeal on TET.

## Supplementary Information

Below is the link to the electronic supplementary material.

Supplementary file1 (DOCX 12 KB)

Supplementary file2 (MP4 12513 KB)

Supplementary file3 (MP4 14166 KB)

## Data Availability

All data generated or analyzed during this study are included in the article. Further enquiries can be directed to the corresponding author.
