# Correction: The Usefulness of AirSeal™ Intelligent Flow System in Gas Insufflation Total Endoscopic Thyroidectomy

**DOI:** 10.1007/s12070-023-04080-x

**Published:** 2023-07-28

**Authors:** Hiroshi Katoh, Yoshifumi Ikeda, Yoshiyuki Saito, Mitsuo Yokota, Mariko Kikuchi, Norihiko Sengoku, Kaoru Fujisaki, Takafumi Sangai

**Affiliations:** 1 Department of Breast and Endocrine Surgery, Kitasato University Hospital, 1-15-1 Kitasato, Minami-ku, Sagamihara, Kanagawa 252-0374 Japan; 2 Department of Surgery, International University of Health and Welfare, Atami Hospital, Atami, Japan; 3 Department of Surgery, Keio University School of Medicine, Tokyo, Japan

**Correction to**: **Indian J Otolaryngol Head Neck Surg 75(1):115–120** 10.1007/s12070-022-03257-0

The article “The Usefulness of AirSeal™ Intelligent Flow System in Gas Insufflation Total Endoscopic Thyroidectomy”, written by Hiroshi Katoh, Yoshifumi Ikeda, Yoshiyuki Saito, Mitsuo Yokota, Mariko Kikuchi, Norihiko Sengoku, Kaoru Fujisaki and Takafumi Sangai., was originally published Online First without Open Access. After publication in volume 75, issue 1, page 115–120 the author decided to opt for Open Choice and to make the article an Open Access publication. Therefore, the copyright of the article has been changed to © The Author(s) 2023 and the article is forthwith distributed under the terms of the Creative Commons Attribution 4.0 International License, which permits use, sharing, adaptation, distribution and reproduction in any medium or format, as long as you give appropriate credit to the original author(s) and the source, provide a link to the Creative Commons licence, and indicate if changes were made. The images or other third party material in this article are included in the article’s Creative Commons licence, unless indicated otherwise in a credit line to the material. If material is not included in the article’s Creative Commons licence and your intended use is not permitted by statutory regulation or exceeds the permitted use, you will need to obtain permission directly from the copyright holder. To view a copy of this licence, visit http://creativecommons.org/licenses/by/4.0.

Original article has been corrected.

