# Supplementary material for: The Usefulness of AirSeal™ Intelligent Flow System in Gas Insufflation Total Endoscopic Thyroidectomy
Source: Indian J Otolaryngol Head Neck Surg. 2022 Nov 6;75(1):115–20. doi: 10.1007/s12070-022-03257-0 (PMC10050613; doi:10.1007/s12070-022-03257-0)
Supplement: Supplementary file 1 — Supplementary file1 (DOCX 12 KB) [file 12070_2022_3257_MOESM1_ESM.docx]

**Legends for videos**

**Video 1. Representative video of total endoscopic right hemi-thyroidectomy without AirSeal system.**

Smoke/mist caused by energy device (Harmonic HD) obstructs visibility and suctioning of smoke/mist narrows working space.

**Video 2. Representative video of total endoscopic right hemi-thyroidectomy in which AirSeal system dramatically improved visibililty.**

AirSeal application dramatically improved visibility by decreasing smoke/mist. AirSeal system prevents narrowing on suctioning by retaining stable pressure as well. Video presentations demonstrate excellent visibility by AirSeal application.
